# Effect of Nanoparticles on the Thermal Stability and Reaction Kinetics in Ionic Nanofluids

**DOI:** 10.3390/nano12101777

**Published:** 2022-05-23

**Authors:** Adela Svobodova-Sedlackova, Sergio Huete-Hernández, Alejandro Calderón, Camila Barreneche, Pablo Gamallo, Ana Inés Fernandez

**Affiliations:** 1 Departament de Ciència de Materials i Química Física, Universitat de Barcelona, C/Martí i Franqués 1, 08028 Barcelona, Spain; adela.svobodova@ub.edu (A.S.-S.); sergio.huete@ub.edu (S.H.-H.); c.barreneche@ub.edu (C.B.); gamallo@ub.edu (P.G.); 2 Institut de Química Teòrica i Computacional, IQTCUB, Universitat de Barcelona, C/Martí i Franqués 1, 08028 Barcelona, Spain; 3 Departament d’Enginyeria Mecànica, Universitat Rovira i Virgili, Av. Paisos Catalans 26, 43007 Tarragona, Spain; acalderon@ub.edu

**Keywords:** nanofluids, thermal stability, nanoparticles, molten salts, UV-vis spectrum, thermogravimetry analysis (TGA), reaction kinetics

## Abstract

Nowadays, the incorporation of nanoparticles into thermal fluids has become one of the most suitable strategies for developing high-performance fluids. An unconventional improvement of thermo–physical properties was observed with the addition of 1% wt. of nanoparticles in different types of fluids, such as molten salts, allowing for the design of more thermally efficient systems using nanofluids. Despite this, there is a lack of knowledge about the effect that nanoparticles produce on the thermal stability and the decomposition kinetics of the base fluid. The present study performs IR- and UV-vis spectroscopy along with thermogravimetric analysis (TGA) of pure nitrate and nitrate based nanofluids with the presence of SiO_2_ and Al_2_O_3_ nanoparticles (1% wt.). The results obtained support that nanoparticles accelerate the nitrate to nitrite decomposition at temperatures below 500 °C (up to 4%), thus confirming the catalytic role of nanoparticles in nanofluids.

## 1. Introduction

The incorporation of suspended nanoparticles (NPs) into a fluid has become a suitable strategy for improving the thermo–physical properties of fluids; this concept, defined as a nanofluid (NF), was first introduced in 1995 by Choi et al. [1], who showed that the thermal conductivity of water and ethylene glycol increased by adding Al_2_O_3_ or CuO NPs; since then, many efforts have been devoted to the development of NFs as well as to the understanding of some exceptional properties exhibited by them [2,3]. More precisely, one of the aspects that attracted the scientific interest is the abnormal improvement of the specific heat capacity (*C_p_*) observed in NFs; thus, a large number of publications indicate *C_p_* increments up to 40% when low concentrations of NPs are incorporated into the fluid [4], around 1% wt. According to this, and to the fact that material’s energy density is determined by the product of *C_p_* and the fluid density, the heat capacity becomes one of the most relevant design parameters in industrial applications allowing to reach more compact and effective heat transfer systems using NFs. Consequently, NFs open the door to the next generation of heat transfer fluids (HTF) with better thermal performance than the traditional fluids such as water, oils, molten salts or ethylene glycol, solving their relatively poor heat transfer characteristics. In the last years, NFs have been incorporated in many applications such as solar energy [5,6], geothermal [7], heat exchange [7], oil recovery [8,9], lubricants [10,11], refrigeration [12], desalination [13] or CO_2_ capture [14], among others.

Specifically, the incorporation of NFs as a thermal energy storage (TES) medium in concentrate solar power (CSP) plants would improve the storage efficiency and thus, it may contribute to the possibility of reducing the volume of the storage tanks, involving an important material cost reduction [15,16,17]. Molten salts and particularly solar salt (i.e., a eutectic mixture of sodium and potassium nitrate) was the most commercially used TES material in CSP plants. Molten salt-based NFs are widely studied in the literature [18,19,20,21], showing a high *C_p_* enhancement. For example, Y. Huang et al. [22] report *C_p_* enhancements up to 168% after adding MgO NPs into the solar salt; however, the majority of publications report *C_p_* enhancements up to 30% adding NPs at different concentrations into molten salts systems like Li_2_CO_3_/K_2_CO_3_ + 1% wt. SiO_2_ [23], NaNO_3_/KNO_3_ + 0.8% wt. Al_2_O_3_ [24], KNO_3_/NaNO_2_/NaNO_3_ + 0.07% wt. Al_2_O_3_ [25], NaNO_3_ + 1% wt. SiO_2_ [4] and Ca(NO_3_)_2_/KNO_3_/NaNO_3_/LiNO_3_ + 0.5% wt. SiO_2_ [26], among others. Nevertheless, some studies show just the opposite trend [27,28,29]; it is the case of the studies carried out by Q. Xie et al. [30], M. A. Hassan et al. [31] or M. C. Lu et al. [32], in which a decrease in *C_p_* was reported by the addition of NPs in molten salts. Hence, there are no clear trends regarding the effect of *C_p_* enhancement due to the addition of nanoparticles in the heat transfer fluid.

Another relevant parameter for the implementation of NFs in industrial applications is their thermal stability (i.e., solar salts for TES in CSP stations work from 250 to 400 °C). Pramod et al. [33] demonstrated that NPs in molten salts help to improve their thermal stability. Contrarily, no significant changes were observed by *p*. Myers et al. [34] and P. Andreu-Cabedo et al. [35] adding CuO or SiO_2_ NPs into molten salts, respectively. The thermal stability of solar salts (a eutectic mixture of NaNO_3_ (60%)-KNO_3_ (40%)) is directly related to the nitrate-nitrite conversion; therefore, more precise experiments are needed to understand the effect of NPs on the kinetics of the thermal decomposition. Likewise, monitoring the decomposition rate of nitrates (i.e., nitrites formation) is essential to control the NF’s stability as the nitrite formation contributes to salt decomposition and to increase corrosion rates in the storage systems; this aspect is very relevant due to the high corrosion caused by salts in the metallic components of CSP facilities [36,37,38].

This work aims at studying the effect of introducing NPs in the sodium nitrate salt thermal stability. For this propose, IR and UV spectroscopic techniques have been used for determining the nitrite concentration in aqueous solution in the temperature range from 100 to 500 °C. Furthermore, thermogravimetric analysis was performed to study the weight loss, temperature decomposition and reaction kinetics through representative samples of two types of NaNO_3_ based NFs (NaNO_3_ + 1% wt. SiO_2_ and NaNO_3_ + 1% wt. Al_2_O_3_) in the same range of temperatures for evaluating the degree of nitrites formation and the temperature stability of the samples; other techniques, such as electron microscopy, were also used for characterizing the NPs.

## 2. Materials and Methods

### 2.1. Nanofluids Sample’s Preparation

**Figure 1:**
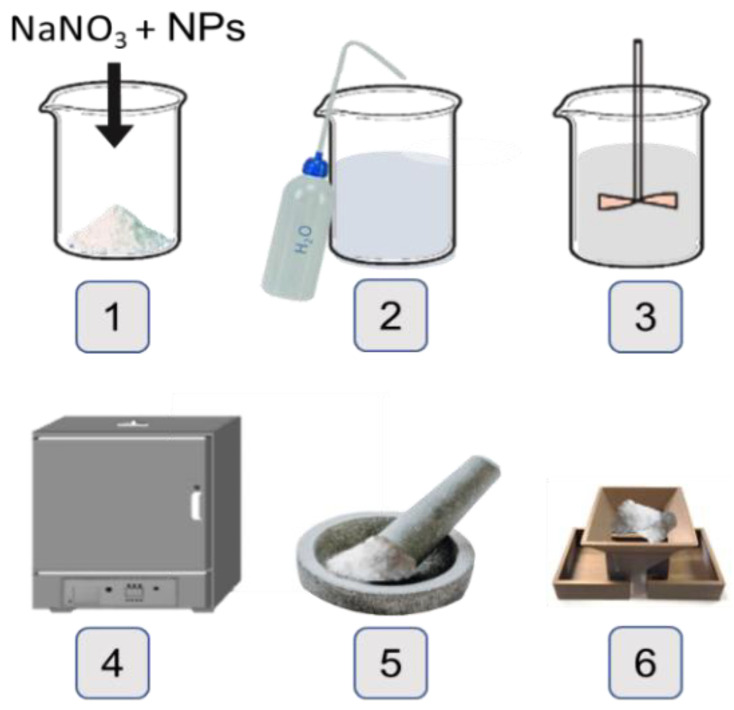
Schematic representation of the two-step nanofluid synthesis.

NaNO_3_ used to synthetize the NFs was Sigma Aldrich (99.995%), spherical SiO_2_ and Al_2_O_3_ NPs of 5–15 nm and 13 nm, respectively, of nominal diameter (both Sigma Aldrich, 99.5%) (Sigma Aldrich, St. Louis, MO, USA). The synthesis of the NFs were carried out through the following six steps (described in Figure 1): (1) weighting of NaNO_3_ and 1% of NPs (wt./wt.); (2) dissolving the mixture in 20 mL of distilled water; (3) sonicating during 20 min for a correct dispersion and homogenization of NPs in the solution; (4) drying in an oven at 105 °C until complete water evaporation and salt recrystallization; (5) grinding in an Agatha mortar; and (6) obtaining a representative sample by following the quartering standard methodology [39] with a hand-made riffle-splitter suitable for tiny amounts of sample.

### 2.2. Transmission Electron Microscopy

A transmission electron microscope (TEM) JEOL JEM 2100 (JEOL, Tokyo, Japan) was employed to characterize the NPs. To proceed, NPs were dispersed in ethanol and sonicated by ultrasonic bath to avoid agglomeration.

### 2.3. UV-Spectroscopy

A UV-Vis Spectrophotometer Shimadzu UV-1280 (Shimadzu, Kyoto, Japan) was employed to measure the absorbance and quantify the nitrite’s ion concentration (i.e., [${\mathrm{NO}}_{2}^{-}]$). The absorbance measurements were performed in the wavelength range between 230 to 600 nm. For this purpose, 10 independent samples were synthesized for each type of NP into pure sodium nitrate. First, each sample was subjected to different thermal treatments in a furnace at room temperature and then heated at intervals of 50 °C from in a temperature range between 50 and 500 °C. For each temperature, the sample was left in the furnace for around 30 min to ensure a homogeneous temperature of the sample. Subsequently, the samples were cooled into liquid nitrogen to freeze the structure at each temperature. After the thermal treatment, 0.3 M solutions were prepared by dissolving the samples in deionized water; moreover, 0.3 M sodium nitrate samples with different concentrations of sodium nitrite (2.5%, 5%, 12.5% and 25% and 100% wt.) were prepared for the nitrite calibration. The measurement uncertainty in the absorbance values was ±0.001 in arbitrary units.

### 2.4. Thermogravimetric Analysis

To perform the thermogravimetric and differential thermogravimetric analysis (TG/DTG), a Q-600 SDT TA Instruments (TA instruments, New Castle, DE, USA) was used. Measurements were conducted from 30 °C to 900 °C at a heating rate of 10 °C min^−^^1^ in air atmosphere with a gas flow of 100 mL min^−^^1^. Each sample was prepared in standard aluminum crucibles with around 11 mg wt. Uncertainties were 0.5 °C for temperature, 1% for weight loss and 0.01% for mass.

### 2.5. pH 

A pH and ion-meter GLP 22 from Crison (Crison Instruments, Alella, Spain) was employed to measure the pH of the samples at room temperature (29.5 ± 0.2 °C) with an uncertainty of 1%.

### 2.6. FT-IR Spectroscopy

Fourier Transform Infrared Spectroscopy with Attenuated Total Reflectance (FT-IR ATR) technique with a spectrometer TwoTM by PerkinElmer (PerkinElmer, Waltham, MA, USA) was used to determine the chemical composition. The instrumental error associated with the measure was 4 cm^−1^.

## 3. Results

### 3.1. Nanoparticle’s Characterization

**Figure 2:**
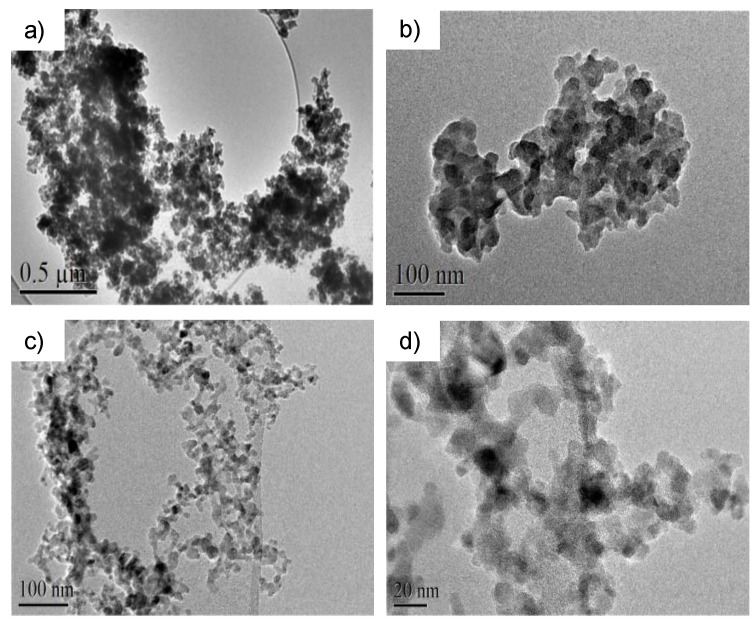
TEM images at different magnifications for SiO_2_, (**a**,**b**), and Al_2_O_3_ nanoparticles, (**c**,**d**).

The size and the concentration of the NPs are important parameters that govern the NF performance [40,41]. The characterization of the nanoparticles has been done through pure samples electronic microscopy. Thus, Figure 2 shows TEM images of the SiO_2_ and Al_2_O_3_ NPs. The silicon oxide NPs exhibit a higher degree of sintering, forming agglomerates of even more than one micrometer (Figure 2a,b). The SiO_2_ nanoparticles’ length and width were around: (12 × 25) nm^2^. On the contrary, alumina NPs have a nominal diameter in the range 10.8–12.3 nm (Figure 2c,d).

### 3.2. Nitrite Determination

**Figure 3:**
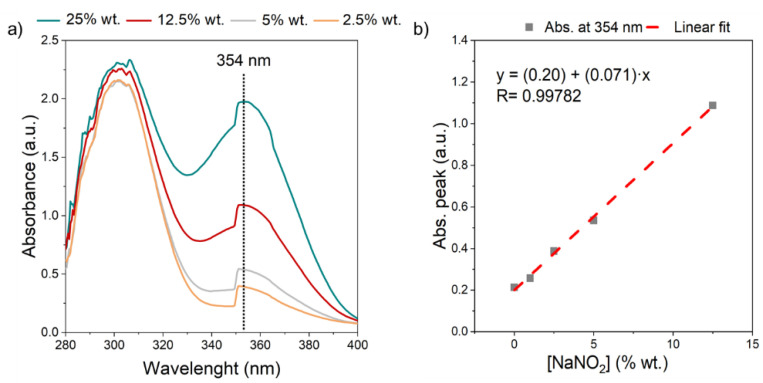
UV-vis spectra of 0.3M sodium nitrate-nitrite solutions: (**a**) evolution of nitrite peak with the concentration of NaNO_2_, from 2.5 to 25% wt. in NaNO_3_ solution, (**b**) linear relationship between light absorption and the concentration of nitrites at a wavelength of 354 nm.

Indirect methods based on UV and visible spectroscopy allow quantifying the nitrite concentration with an excellent limit of detection [42,43]. The UV-Vis absorption spectra were performed to NaNO_3_/NaNO_2_ mixtures samples for 0.3 M (in deionized water) at 2.5%, 5%, 12.5% and 25% wt. in NaNO_2_ concentration_,_ Figure 3. The characteristic absorption peaks for [${\mathrm{NO}}_{2}^{-}]$ and [${\mathrm{NO}}_{3}^{-}]$ ions were found at 354 nm and 300 nm, respectively [43,44]. The most interesting result is the increase observed in the nitrite peak as the concentration increases from 2.5% to 25% wt., Figure 3a. The absorbance of nitrite peaks as a function of concentration (Figure 3b) exhibits a perfect linear trend with R = 0.99782, a = 0.19999 ± 0.01013 (intersection) and b = 0.0706 ± 0.00165 (slope) with a good accuracy, σ = ± 0.2.

**Figure 4:**
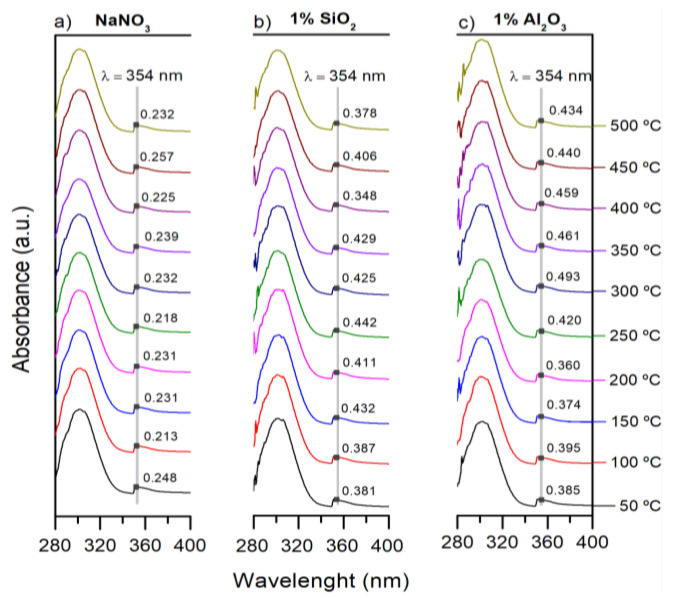
UV-vis absorption spectra of samples after the thermal treatment from 50 to 500 °C: (**a**) pure 0.3M NaNO_3_, (**b**) 0.3M NaNO_3_/SiO_2_ NF (1% wt.) and (**c**) 0.3M NaNO_3_/Al_2_O_3_ NF (1% wt.).

**Figure 5:**
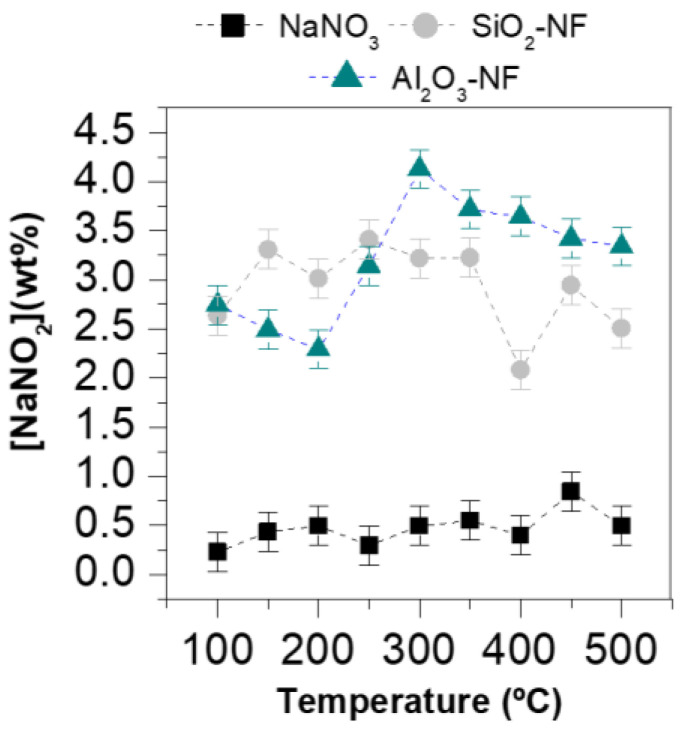
Nitrite concentration [NaNO_2_] as a function of temperature from 100 to 500 °C for pure 0.3M NaNO_3_ (black symbols), 0.3M NaNO_3_/SiO_2_ NF (1% wt.) (grey symbols) and 0.3M NaNO_3_/Al_2_O_3_ NF (1% wt.) (green symbols).

Samples were exposed to different thermal treatments, from room temperature to 500 °C and then cooled in liquid N_2_ to freeze the structure at each temperature. Figure 4 shows the temperature evolution of the UV-Vis spectrum of pure 0.3M NaNO_3_ (Figure 4a), 0.3M NaNO_3_/SiO_2_ NF (Figure 4b), and 0.3M NaNO_3_/Al_2_O_3_ NF (Figure 4c); moreover, for each spectrum, the absorbance intensity at 354 nm was determined (grey line). For the pure NaNO_3_ and the two formulated NFs, an intensity difference in the nitrite peak with temperature was observed. Thus, Table 1 summarizes all the absorbance intensities at 354 nm as a function of the temperature; additionally, for each value, the predicted NaNO_2_ concentration was determined by the linear fit of Figure 3b. Both formulated NFs show a slight increase in the concentration of NaNO_2_ (absorbance of 0.378 and 0.434 for SiO_2_ and Al_2_O_3_ NPs at 1% wt. at 50 °C) in front of the pure NaNO_3_ (absorbance of 0.232 at 50 °C) caused by the presence of NPs. Therefore, the NPs modify the reaction kinetics of NaNO_3_ decomposition in the temperature range explored; moreover, Al_2_O_3_ NPs accelerate the nitrate-nitrite conversion even more than the SiO₂ NPs. This fact indicates that the nature of the NPs influences the chemical degradation of NFs, and thus, the more alkaline NPs (Al_2_O_3_ > SiO_2_) the greater variation in absorbance for the temperature range; however, it is remarkable that the maximum concentration of NaNO_2_ was around 300 °C for the 2 NFs. Despite this, it is noteworthy that the maximum NaNO_2_ concentration was less than 4 ± 0.2% for the two NFs and less than 0.8 ± 0.2% for the pure NaNO_3_, Figure 5.

Nonetheless, the nitrite concentration in the aqueous solution is strongly pH-dependent. At pH values higher than 5 (pH > 5), the literature suggests that the UV absorbance have good linearity with nitrite concentration [45]. To corroborate the adjustment and then, the nitrite concentration, the pH was measured for all the samples of Table 1 and the results are summarized in Table 2. The pH values were in the range 5–7; furthermore, with the addition of both NPs, the acidity of the NFs slightly decreases (i.e., the pH increases). Since the pH of the dissolution of nitrite was 7.08 ± 0.01, the increase of pH in the two formulated NFs was in accordance with the increase of NaNO_2_ concentration in the samples obtained from the linear fit in Table 1.

**Figure 6:**
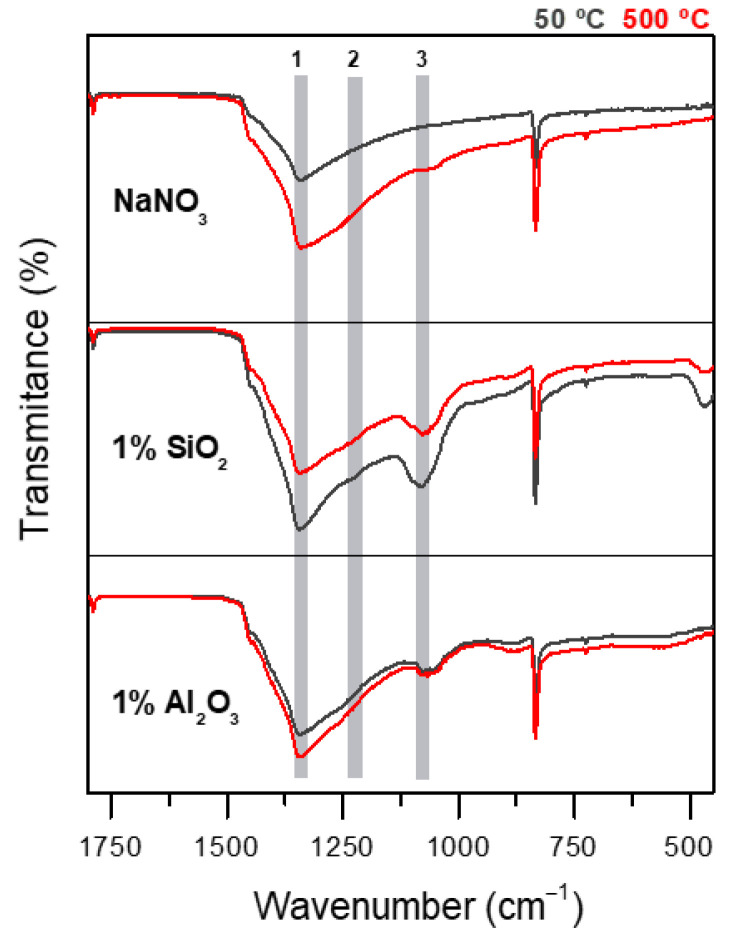
FT-IR spectra of NaNO_3_ and NaNO_3_/SiO_2_, NaNO_3_/Al_2_O_3_ NFs with 1% wt. of NPs after thermal treatment at 50 °C and 500 °C. Band (1) corresponds to asymmetric stretching mode of ${\mathrm{NO}}_{3}^{-}$, and bands (2)–(3) corresponds to fundamental vibration bands of ${\mathrm{NO}}_{2}^{-}$.

To corroborate and validate the nitrite formation, FT-IR spectroscopy was employed to determine the chemical composition of the samples. The FT-IR spectra for pure NaNO_3_ and the two NFs after thermal treatments at 50 °C and 500 °C, are shown in Figure 6. There are several vibrational bands that allow to identify the NaNO_3_-NaNO_2_ conversion, three of them are: (1) the relative intensity of the band at 825 cm^−1^ corresponding to the bending mode ν_2_ of ${\mathrm{NO}}_{2}^{-}.$ The relative intensity of this mode decreases with the increase of [${\mathrm{NO}}_{2}^{-}]$ also observing a slight shift of the mode frequency from 835 cm^−1^ to 825 cm^−1^. (2) Modification of the band contour of the ν_4_ mode, 725 cm^−1^, that corresponds to the asymmetric in-plane bending mode; this band begins to appear as the [${\mathrm{NO}}_{3}^{-}]$ decrease for x = 0.6 ($\frac{\left[{\mathrm{NO}}_{2}^{-}\right]\;}{[{\mathrm{NO}}_{3}^{-}]\;}$) and exhibits a slight shift from 725 cm^−1^ to 715 cm^−1^. (3) The narrowing of the band located around 1358 cm^−1^, corresponding to the asymmetric stretching mode of [${\mathrm{NO}}_{3}^{-}],$ and the presence of the band at 1271 cm^−1^, corresponding to one of the fundamental vibrational modes of NaNO_2_, ν_3_ [46,47]. Nonetheless, at low [${\mathrm{NO}}_{2}^{-}]$ concentrations, band (3) is the only that can monitor the evolution of nitrite concentration [48,49]. Figure 5 shows the 3 bands: (1) ~1358 cm^−1^, (2) ~1270 cm^−1^, and (3) ~1100 cm^−1^. All the samples, except for NaNO_3_, show the presence of the tiny band (2) and a slight broadening of the band (1) as temperature increases. The relative intensity of the band (2) is increased in the NFs samples, indicating a high nitrite formation in comparison to pure NaNO_3_. The low intensity of this band agrees with the low [${\mathrm{NO}}_{2}^{-}]$ concentrations determined in Table 1; additionally, the presence of the band (3) in the NaNO_3_ sample at 500 °C, is a good indicator of the presence of low [${\mathrm{NO}}_{2}^{-}]$ concentrations, between x = 0.1/0.2 [48,49,50]. In the NFs spectra this band appears with higher intensity corroborating the [${\mathrm{NO}}_{2}^{-}]$ formation. Nonetheless, in the case of SiO_2_ NFs, this band overlaps with the asymmetric stretching of SiO_2_, causing a higher band intensity.

### 3.3. Nanofluids Reaction Kinetics and Decomposition

**Figure 7:**
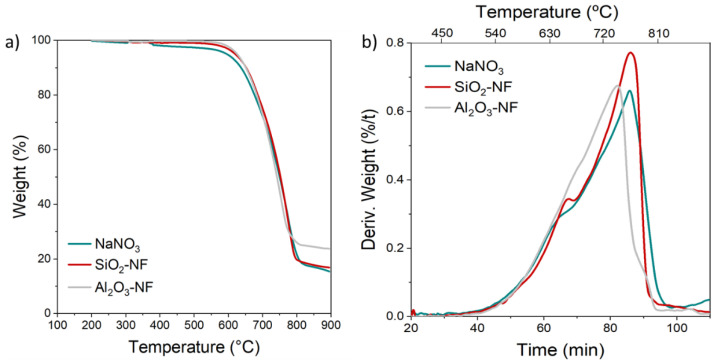
TGA measurement from 100 to 900 °C of NaNO_3_, and NaNO_3_/SiO_2_, NaNO_3_/Al_2_O_3_ NFs with 1% wt. of NPs: (**a**) weight loss as a function of temperature and, (**b**) weight derivative as a function of time and temperature.

The increase of NaNO_2_ concentration in the NFs indicates a change in the reaction kinetics of NaNO_3_ decomposition. To study the effect of the NPs in the decomposition of NaNO_3_, the sample weight loss and derivative weight over time from 100 to 900 °C were evaluated in Figure 7. In the temperature range between 100 and 500 °C, Figure 7a, no significant weight loss was observed for pure NaNO_3_ and NaNO_3_/SiO_2_, NaNO_3_/Al_2_O_3_ NFs. The maximum weight loss was 2.5% in the case of pure NaNO₃ and lower than 1% for the two formulated NFs. Hence, NFs were slightly more stable than the pure NaNO_3_. On the other hand, due to the limit of detection (1%) in the weight loss, it is not possible to identify the low NaNO_3_-NaNO_2_ conversion determined in Table 1. Conversely, the NFs samples did not show any relevant change in the temperature range sampled, and they start to decompose around 600 °C, as previously suggested for pure NaNO_3_ [51]. It is remarkable that after the total decomposition, ≈800 °C, the weight loss of Al_2_O_3_-NF was lower than SiO₂ NF and pure NaNO_3._

Figure 7b, shows the derivative of the weight (d(%/t)), as a function of time. The derivative of weight loss gives information of the reaction kinetics. Dissimilar behaviours were identified. Two main peaks were observed for the three samples, approximately between 278–699 °C (around 60 min) and at 730–775 °C (between 80–86 min), respectively. On the other hand, a third peak was observed for the AlO_3_ NF sample over 795 °C (around 90 min) The principal decomposition peak (around 80 min) shows a variation in time and temperature. Consequently, a variation in the reaction kinetics and the thermal stability of the salt (i.e., the equilibrium constants and the activation energy) are modified with the addition of AlO_3_ and SiO_2_ NPs. Table 3 summarizes all the main identified temperature peaks and the associated weight loss.

**Figure 8:**
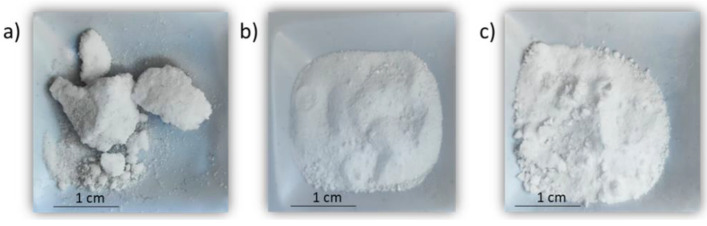
Effect of moisture on physical properties (water absorption) of (**a**) pure NaNO_3_, (**b**) NaNO_3_/SiO_2_ NPs (1% wt.) and (**c**) NaNO_3_/Al_2_O_3_ NPs (1% wt.).

The lowest weight loss before the beginning of the decomposition above 600 °C can be explained by the lower hygroscopicity of NFs; the NPs help to reduce the moisture absorption. Figure 8 illustrates the change that experiments sodium nitrate after one year of exposure at room temperature when it is pure (a) and in the presence of SiO_2_ (b) and Al_2_O_3_ (c) NPs. Pure NaNO_3_ showed more aggregates than sodium nitrate with the presence of NPs due to moisture absorption; this behaviour is a relevant finding on the point of view of molten salts application because their high hygroscopicity in contact with environment increases the damage of metal compounds present in the plant [52,53]; thus, this effect is reduced considerably with the presence of NPs.

To better understand the thermal decomposition and to relate the TGA profiles to the possible reactions involved, the deconvolution of the TGA peaks have been statistically performed by a Gaussian fit [54]. A total of 15 reactions have been taken into consideration corresponding all to nitrate-nitrite conversions and to sodium oxides decompositions:

(1) $${\mathrm{NaNO}}_{3\left(s\right)}⇋{\mathrm{NaNO}}_{2\left(s\right)}+\frac{1}{2}O_{2(g)}$$

(2) $${\mathrm{NO}}_{3\left(l\right)}^{-}⇋{\mathrm{NO}}_{2\left(l\right)}^{-}+\frac{1}{2}O_{2\left(g\right)}$$

(3) $${\mathrm{NO}}_{3\left(l\right)}^{-}+\frac{1}{2}O_{2\left(g\right)}⇋{\mathrm{NO}}_{2\left(l\right)}^{-}+O_{2\left(g\right)}$$

(4) $${\mathrm{NaNO}}_{2\left(l\right)}\to \frac{1}{2}{\mathrm{Na}}_{2}O_{\left(s\right)}+\mathrm{NO}+\frac{1}{4}O_{2\left(g\right)}$$

(5) $${\mathrm{NaNO}}_{2\left(l\right)}⇋\frac{1}{2}{\mathrm{Na}}_{2}O_{2\left(s\right)}+{\mathrm{NO}}_{\left(g\right)}$$

(6) $$2{\mathrm{NaNO}}_{3\left(l\right)}\to {\mathrm{Na}}_{2}O_{\left(l\right)}+{\mathrm{NO}}_{2\left(g\right)}+{\mathrm{NO}}_{\left(g\right)}+O_{2\left(g\right)}$$

(7) $$2{\mathrm{NaNO}}_{2\left(l\right)}+2{\mathrm{NO}}_{\left(g\right)}\to 2{\mathrm{NaNO}}_{3\left(l\right)}+N_{2\left(g\right)}$$

(8) $${\mathrm{NaNO}}_{2\left(l\right)}+{\mathrm{NO}}_{2\left(g\right)}\to {\mathrm{NaNO}}_{3\left(l\right)}+{\mathrm{NO}}_{\left(g\right)}$$

(9) $${\mathrm{Na}}_{2}O_{2\left(s\right)}+2{\mathrm{NO}}_{2\left(g\right)}\to {\mathrm{NaNO}}_{3\left(l\right)}+{\mathrm{NaNO}}_{2\left(l\right)}+\frac{1}{2}O_{2\left(g\right)}$$

(10) $${\mathrm{NaNO}}_{2\left(l\right)}⇋\frac{1}{2}{\mathrm{Na}}_{2}O_{\left(s\right)}+{\mathrm{NO}}_{\left(g\right)}+\frac{1}{4}O_{2\left(g\right)}$$

(11) $${\mathrm{Na}}_{2}O_{2\left(s\right)}\to {\mathrm{Na}}_{2}O_{\left(s\right)}+\frac{1}{2}O_{2(g)}$$

(12) $${\mathrm{Na}}_{2}O_{2\left(s\right)}+\frac{1}{2}O_{2\left(g\right)}⇋2{\mathrm{NaO}}_{\left(s\right)}+\frac{1}{2}O_{2\left(g\right)}$$

(13) $${\mathrm{Na}}_{2}O_{2\left(s\right)}⇋{\mathrm{Na}}_{2}O_{\left(s\right)}+\frac{1}{2}O_{2\left(g\right)}$$

(14) $$2{\mathrm{NaNO}}_{2\left(l\right)}⇋{\mathrm{Na}}_{2}O_{\left(s\right)}+N_{2}+\frac{3}{2}O_{2}$$

(15) $$2{\mathrm{NaNO}}_{3\left(l\right)}⇋{\mathrm{Na}}_{2}O_{\left(s\right)}+N_{2\left(l\right)}+\frac{5}{2}O_{2\left(g\right)}$$

**Figure 9:**
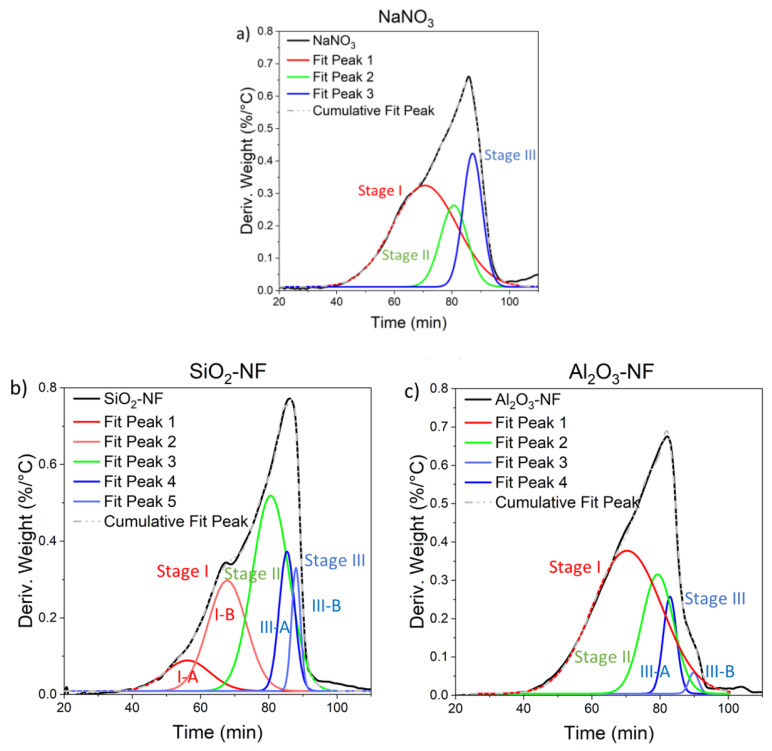
Non-linear peak deconvolution fit of the TGA weight derivative over time for (**a**) NaNO_3_, (**b**) NaNO_3_/SiO_2_ NF (1% wt.) and (**c**) NaNO_3_/Al_2_O_3_ NF (1% wt.).

Figure 9 shows the TGA peak deconvolution and the cumulative fit peak (grey dotted line) for pure NaNO₃ (Figure 9a), for SiO₂ NF (Figure 9b) and AlO₃ NF (Figure 9c). All the Gaussian models (cumulative fit peak) show a good fitting (i.e., R^2^ = 0.996, 0.998 and 0.999 for pure NaNO_3_, SiO_2_ and Al_2_O_3_ NFs, respectively); moreover, Table 4 summarizes the parameters of the deconvoluted peaks and also the reactions that contribute to each of them.

Three main stages were identified in the peak deconvolution of NaNO_3_ in Figure 9a. The decomposition of NaNO₃ has been well studied in the literature [55,56,57,58,59], and it is known that at high temperatures as a result of its decomposition, nitrite and sodium oxides species coexist; moreover, the decomposition process of nitrate salt can be subdivided into several stages that occur simultaneously and/or consecutively.

**Stage I**: The first identified stage corresponds to the solid-state reaction of formation of NaNO_2_, above the melting temperature to 450 °C, according to Equation (1). The equilibrium of this reversible reaction depends on the temperature and, above 600–730 °C, the backward reaction with oxygen is slower than the decomposition [55], Equation (2). In Stage I-B occurs the oxidation-decomposition processes, Equations (2) and (3); this peak reaches the maximum value at 697.3 ± 0.5 °C (at a velocity of 9.88 ± 0.01 °C/min) for pure NaNO_3_. Noticeably, the same peak for SiO_2_ NF (Figure 9b) and for Al_2_O_3_ NF (Figure 9c) appears shifted at lower temperatures (i.e., −14.3 ± 0.7 °C and −1.6 ± 0.7 °C, respectively). Furthermore, the addition of SiO_2_ NPs make that the single peak decomposes into two peaks (I-A and I-B), varying the decomposition kinetics of Equations (1)–(3). Specifically, an initial decomposition stage appears, Stage I-A, with a maximum at 625.8 ± 0.5 °C, with highest reaction kinetics (11.1 ± 0.1 °C/min); therefore, the addition of NPs drives to an acceleration of decomposition at lower temperatures than pure NaNO_3_ (Table 3).

**Stage II**: In the intermediate stage, from 450 to 700 °C, the first-order liquid-liquid reaction occurs, the reverse reaction in the melt sodium nitrite to form sodium nitrate (oxidation-decomposition) Equation (2); this reverse equation is possible due to the formation of NaO₂ or Na_2_O_2_ as intermediates during the decomposition of sodium nitrite, Equations (4)–(10). When the equilibrium of Equation (2) was displaced to the decomposition, the formation of O_2_ and NO is favoured. Therefore, the Equation (4) becomes the most kinetically and energy-favoured reaction [56]. A single peak was identified for the three samples in this stage. The maximum of this stage for NaNO_3_ occurs at 747.9 ± 0.5 °C. A decrease of temperature was observed only with Al_2_O_3_ NPs (−9.4 ± 0.5 °C). Therefore, the presence of Al_2_O_3_ NPs drives to a NaNO_2_ decomposition, Equations (4)–(10), at lower temperatures.

**Stage III**: Finally, above 700 °C, a reaction of NaNO_2_ and direct decomposition of NaNO_3_ occurs with the formation of Na_2_O and release of nitrogen oxides [55], Equations (11)–(15). In the case of pure NaNO_3_ the reaction occurs with a single step, stage III-A, with a maximum at 781.2 ± 0.5 °C. The presence of this peak confirms that Na_2_O_2_ and/or NaO_2_ are formed as intermediates for the formation of Na_2_O, Equations (11)–(15), [60]. In contrast, the decomposition to Na_2_O for NFs follow two steps: III-A and III-B described by parallel reactions. The initial decomposition stage for NFs, III-A, starts at lower temperature than the pure NaNO_3_ (and at lower times). The peak temperature decreases by −9.8 ± 0.7 °C and −22.3 ± 0.7 °C with SiO_2_ and Al_2_O_3_ NPs, respectively, in comparison to pure NaNO_3_. Contrarily, in the final reaction process, III-B, the maximum peak temperature increases +4.2 ± 0.7 °C with SiO_2_ NPs and 13.9 ± 0.7 °C with Al_2_O_3_ NPs.

Consequently, the presence of NPs produces an acceleration of the decomposition of nitrates through the sequence NaNO_3_-NaNO_2_-Na_2_O_2_, and a shift to high temperatures in the formation of Na_2_O. Despite the final decomposition occurs at higher temperatures, it occurs at lower times. Accordingly, the NFs need higher temperatures and lower times to decompose than the pure salt confirming the role of NPs as catalysts of decomposition reactions.

**Figure 10:**
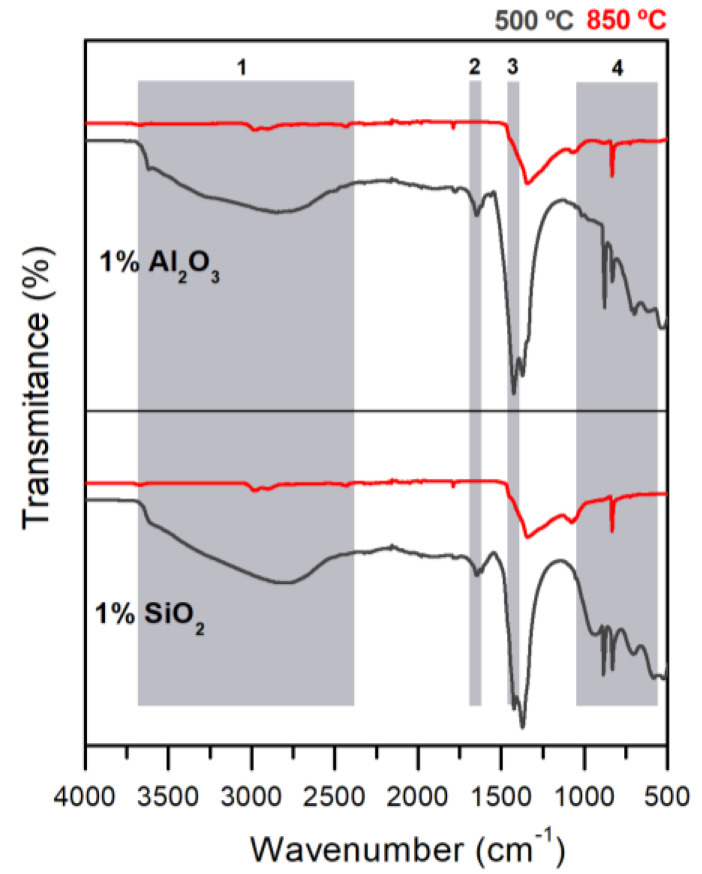
FT-IR spectra for (**top**) NaNO_3_/Al_2_O_3_ NF (1% wt.) and (**bottom**) NaNO_3_/SiO_2_ NF (1% wt.) after thermal treatment at 500 °C and 850 °C.

Moreover, some authors described a chemical reaction between NaNO_3_ and NPs, generating new intermediate species like Na_2_SiO_3_ [61,62]. To corroborate the reactivity between the NPs and NaNO_3_, the final product of the two NFs after the thermal treatment (after stage III, 850 °C) was analysed by FT-IR. Figure 10, shows the comparison between FT-IR spectra at 500 °C and 850 °C, for the two NFs. The identified bands at the regions (1–4), indicate the formation of new species. In the case of presence of Al_2_O_3_ NPs, these bands indicated the formation of species like NaAlO_2_ [63]. Similarly, the presence of SiO_2_ NPs favours the formation of NaSiO_2_. The bands in the region (1) and (4) indicate the mixed formation of NaSiO_2_ and Na_2_SiO_3_ [64]; this fact can explain the variation in decomposition rates and temperatures, as demonstrated by the study carried out by Y. Hoshino et al. [49], with the addition of several oxides at the μm scale into NaNO_3_.

## 4. Conclusions

The effect of the incorporation of nanoparticles on the thermal stability of NaNO_3_ was investigated in this study. Through UV-Vis spectroscopy and thermogravimetric analysis, NaNO_2_ concentration and decomposition were studied for pure NaNO_3_ and two NaNO_3_-based nanofluids formulated with 1% wt. of SiO_2_ and Al_2_O_3_ nanoparticles, respectively. Three key findings were achieved:

1. Detection of higher nitrite concentration (up to 4% wt.) than pure NaNO_3_ (up to 0.8% wt.), due to the presence of nanoparticles in the temperature range from 50 °C to 500 °C. Al_2_O_3_ nanoparticles cause a higher nitrate-nitrite conversion than SiO_2_ nanoparticles.
2. The presence of nanoparticles increases thermal stability to over 600 °C before starting to decompose. In addition, with Al_2_O_3_ nanoparticles, weight loss at 900 °C was about 6% lower than NaNO_3_.
3. Three main reaction stages were identified in the NaNO_3_ decomposition in accordance with the literature. These decomposition stages are altered by the presence of nanoparticles. First, SiO_2_ and Al_2_O_3_ nanoparticles reduce the decomposition temperatures of NaNO_3_-NaNO_2_-Na_2_O_2_ up to 7 °C. Even so, the reactions involved were accelerated by the presence of nanoparticles. Particularly, SiO_2_ NPs accelerate the reactions more than Al_2_O_3_ NPs. Second, the final decomposition to Na_2_O occurs at higher temperatures (up to 14 °C) than pure NaNO_3_. Nonetheless, the final decomposition takes place in shorter times.

In view of these results, the nanoparticles act as catalyst for the reactions; however, at temperatures above 500 °C, nanofluids exhibit higher thermal stability than pure NaNO_3_ despite the slight increase in nitrite concentration. To conclude, this study demonstrates the adequacy of the use of UV-Vis absorption and deconvolution of the TGA signal to study nitrite concentration and reaction kinetics.

## Figures and Tables

**Table 1:** Nitrite concentration (% wt.) in pure 0.3M NaNO_3_, 0.3M NaNO_3_/SiO_2_ NF (1% wt.) and 0.3M NaNO_3_/Al_2_O_3_ NF (1% wt.) derived from the absorbance (arbitrary units) at 354 nm as a function of temperature from 100 °C to 500 °C.

Sample	NaNO_3_		NaNO_3_/SiO_2_ NF		NaNO_3_/Al_2_O_3_ NF	
Temperature	Abs. at 354 nm	$\left[\;{\mathrm{NaNO}}_{2}\right]$	Abs. at 354 nm	$\left[\;{\mathrm{NaNO}}_{2}\right]$	Abs. at 354 nm	$\left[\;{\mathrm{NaNO}}_{2}\right]$
(°C)	a.u ± 0.001	% wt. ± 0.2	a.u ± 0.001	% wt. ± 0.2	a.u ± 0.001	% wt. ± 0.2
100	0.213	0.2	0.387	2.6	0.395	2.7
150	0.231	0.4	0.432	3.3	0.374	2.5
200	0.232	0.5	0.411	3.0	0.360	2.3
250	0.218	0.3	0.442	3.4	0.420	3.1
300	0.232	0.5	0.425	3.2	0.493	4.1
350	0.239	0.5	0.429	3.2	0.461	3.7
400	0.225	0.4	0.348	2.1	0.459	3.6
450	0.257	0.8	0.406	2.9	0.440	3.4
500	0.232	0.5	0.378	2.5	0.434	3.3

**Table 2:** pH values at room temperature for pure 0.3M NaNO_3_, 0.3M NaNO_3_/SiO_2_ NF (1% wt.) and 0.3M NaNO_3_/Al_2_O_3_ NF (1% wt.) at different thermal treatments from 50 to 500 °C.

Thermal Treatment (°C)	NaNO_3_ pH ± 0.01	NaNO_3_/SiO_2_ NF pH ± 0.01	NaNO_3_/Al_2_O_3_ NF pH ± 0.01
50	5.76	5.52	6.08
100	5.83	6.25	6.18
150	5.92	5.73	6.17
200	5.77	5.51	6.34
250	5.78	5.98	6.31
300	5.99	6.03	6.52
350	5.78	6.42	6.84
400	6.08	6.67	6.74
450	5.88	6.88	6.81
500	5.93	6.96	6.69

**Table 3:** Temperature and weight loss obtained by TGA measurements of pure NaNO_3_ and NaNO_3_ with SiO_2_ and Al_2_O_3_ NFs at 1% wt.

Sample	NaNO_3_	SiO_2_	Al_2_O_3_
Mass (mg) ± 0.01	13.74	14.27	14.55
First peak temperature (°C) ± 0.5	678.7	681.1	698.9
Weight loss at first peak (%) ± 1	18	20	27
Second peak temperature (°C) ± 0.5	774.8	775.2	755.0
Weight loss at second peak (%) ± 1	62	62	45
Third peak temperature (°C) ± 0.5	-	-	794.5
Weight loss at third peak (%) ± 1	-	-	3
Total weight loss between 507–840 °C (%) ± 1	80	81	75

**Table 4:** Gaussian non-linear fit parameters and the predominant reactions involved of the deconvoluted peaks for NaNO_3_, NaNO_3_/SiO_2_ NF (1% wt.) and NaNO_3_/Al_2_O_3_ NF (1% wt.).

Stage	Step	Fit Max. Peak	NaNO_3_	NaNO_3_/SiO_2_ NF	NaNO_3_/Al_2_O_3_ NF	Reactive Processes
			Value ± Std. Dev.	Value ± Std. Dev.	Value ± Std. Dev.	
Stage I	I-A	Peak		Peak 1		(1) (2) (3)
		Time (min.)	-	56.2 ± 0.5	-	
		Temp. (°C)	-	625.8 ± 0.5	-	
	I-B	Peak	Peak 1	Peak 2	Peak 1	
		Time (min.)	70.56 ± 0.06	67.80 ± 0.06	70.34 ± 0.03	
		Temp. (°C)	697.3 ± 0.5	683.0 ± 0.5	695.7 ± 0.5	
Stage II		Peak	Peak 2	Peak 3	Peak 2	(4) (5) (6) (7) (8) (9) (10)
		Time (min.)	80.66 ± 0.15	80.55 ± 0.07	79.28 ± 0.05	
		Temp. (°C)	747.9 ± 0.5	747.0 ± 0.5	740.5 ± 0.5	
Stage III		Peak	Peak 3	Peak 4	Peak 3	(11) (12) (13) (14) (15)
	III-A	Time (min.)	87.17 ± 0.04	85.32 ± 0.05	82.876 ± 0.009	
		Temp. (°C)	781.2 ± 0.5	771.4 ± 0.5	758.9 ± 0.5	
		Peak		Peak 5	Peak 4	
	III-B	Time (min.)	-	87.966 ± 0.008	89.86 ± 0.03	
		Temp. (°C)	-	785.4 ± 0.5	795.2 ± 0.5	
